# Microbial and Antimicrobial Resistance Profiles of Microbiota in Common Carps (*Cyprinus carpio*) from Aquacultured and Wild Fish Populations

**DOI:** 10.3390/ani11040929

**Published:** 2021-03-25

**Authors:** Modestas Ruzauskas, Julija Armalytė, Eglė Lastauskienė, Rita Šiugždinienė, Irena Klimienė, Raimundas Mockeliūnas, Elena Bartkienė

**Affiliations:** 1 Institute of Microbiology and Virology, Faculty of Veterinary Medicine, Lithuanian University of Health Sciences, Mickevičiaus 9, LT-44307 Kaunas, Lithuania; rita.siugzdiniene@lsmuni.lt (R.Š.); irena.klimiene@lsmuni.lt (I.K.); raimundas.mockeliunas@lsmuni.lt (R.M.); 2 Department of Biochemistry and Molecular Biology, Institute of Biosciences, Life Sciences Center, Vilnius University, Sauletekio 7, LT-10257 Vilnius, Lithuania; julija.armalyte@gf.vu.lt; 3 Department of Microbiology and Biotechnology, Institute of Biosciences, Life Sciences Center, Vilnius University, Sauletekio 7, LT-10257 Vilnius, Lithuania; egle.lastauskiene@gf.vu.lt; 4 Institute of Animal Rearing Technologies, Faculty of Animals Sciences, Lithuanian University of Health Sciences, Mickevičiaus 9, LT-44307 Kaunas, Lithuania; elena.bartkiene@lsmuni.lt

**Keywords:** aeromonas, antibiotics, bacteria, bacterial communities, fish microbiome lakes

## Abstract

**Simple Summary:**

This study was focused on differences in microbial varieties in common carp living in two different environments: open fish ponds and in nature. The results demonstrated that wild fish carry more than 2.5 times the bacterial species in their gut compared with aquacultured fish. More than 400 species of bacteria were identified, the majority of which are considered beneficial microbiota. Besides bacterial variety, it was determined that aquacultured fish harbored more bacteria that are treated as pathogens in animals and humans. The frequency of antimicrobial resistance in bacterial indicators was more common in aquacultured fish compared with bacteria from a wild population, therefore fish farming can be treated as a potential source of environmental contamination with antimicrobial resistant bacteria.

**Abstract:**

In this study we analyzed differences in microbial composition and antimicrobial resistance profiles in common carp living in two different environments: fish ponds, where carp have been kept under the same growing conditions over the last 50 years, and from the wild. The results demonstrated that wild fish carry a great variety of bacterial species (448 species with a prevalence of at least 0.01% from the total number of reads). Aquacultured individuals harbored 2.56 times fewer species in their gut. Significant microbial differences were observed in all taxonomic ranks, including bacterial classes and phyla. Besides bacterial variety, it was determined that aquacultured fish harbored more bacteria that are considered pathogens or opportunistic pathogens, such as Moraxellaceae, Flavobacteriaceae, and Staphylococcaceae. The frequency of antimicrobial resistance in bacterial indicators was more common in aquacultured fish than in wild fish, therefore fish farming may be a potential source of environmental contamination with antimicrobial resistant bacteria.

## 1. Introduction

Aquaculture is the farming of fish and other aquatic organisms for food and other purposes. The rapid development of the aquaculture industry in recent years and the increase in the intensity of production has raised questions regarding potential environmental impacts [1]. Aquaculture systems mirror agriculture, in that some aquaculture operations convert land into ponds to grow aquatic organisms, just as land is converted to grow row crops in agriculture [2]. Fish farm effluent not only affects the area immediately surrounding the farm, but can also alter different ecosystems [3]. Effects include the reduction of biomass, density, and diversity of benthos, plankton, and nekton [3].

Aquaculture systems are classified according their location (water-based systems, land-based systems, and integrated farming systems), methods of confinement (closed systems with or without connection to the environment, open ponds), and feed/fertilizer input. Although during recent years aquaculture knowledge has advanced in areas such as water quality, disease control, and generated stock improvements by selective breeding, hybridization, and molecular genetic technologies [4], some technologies have not changed much in decades.

Bacteria are one of the most important components of ecosystems in nature, including inside animals, humans, and plants. They are crucially important for macroorganisms, particularly their digestive systems, as they participate in the digestive process and help to absorb food. Microorganisms within the intestinal tract have been shown to interact with the gut–brain axis, a bidirectional communication system between the gut and the brain, mediated by hormonal, immune, and neural signals. Through these interactions, the microbiota might affect behaviors, including feeding behavior, digestive/absorptive processes (e.g., by modulating intestinal motility and the intestinal barrier), metabolism, as well as the immune response, with repercussions for energy homeostasis and the health of the host [5].

In Lithuania pond-based methods used for growing common carp are similar to those of 40–50 years ago; the same ponds used in the 1960s are still being used now. We suggest that such permanent processes have not only influenced the composition and variety of microbiota in aquacultured fish, but also had negative impact on the surrounding environment. It is known that different diseases, including parasitic, bacterial, viral, and fungal diseases, are often prevalent in aquacultured fish. Globally, more than 13 bacterial genera have been reported to cause bacterial diseases in the aquaculture industry [6]. These pathogens also can be transferred to free-living populations of fish. It is unclear, however, which microbiota can be treated as probiotic in fish and whether they can protect wild fish from pathogenic agents.

New technologies such as next generation sequencing allow microbial diversity to be explored efficiently, comparing microbial populations among different hosts and bodies of water [7]. The other reason to investigate the composition of microbiota, particularly those prevalent in farming systems, is the common usage of antimicrobials for farmed animal treatment, including fish. Although current legislation requires the monitoring of the use of antimicrobials in humans and animals, there is still no information regarding the amount of antimicrobial usage in fish farms in many countries. Two classes of antimicrobials, tetracyclines and amphenicols, are authorized for fish treatment in Lithuania, although there are no data regarding their usage at a country level.

Moreover, in some pond systems, farm-animal manure can be used to promote eutrophication, and this may contain antimicrobial resistant microorganisms and their genes.

Our previous investigations demonstrated that farm animals carry microorganisms resistant to different classes of antibiotics. The aim of this study was to compare bacterial composition and variety in aquacultured and wild common carp (*Cyprinus carpio*), as well as to evaluate differences in antimicrobial resistance patterns of the selected indicator bacteria between the populations.

## 2. Materials and Methods

### 2.1. Place and Samples

Common carp (*Cyprinus carpio*) were caught using fishing rods from aquacultured fish ponds (54°22′9.82″, 23°39′1.74″) (WGS) and from a population of wild carp in a lake in Alytus county, Lithuania (54°25′35.96″, 23°54′51.16″) (WGS) during the summer of 2020. The complexes of ponds varied between 0.4 to 40 ha and stocked multiple age classes of carp. The ponds had been established and used for about 50 years. Although the pond from which the carp were obtained was used for monoculture (*C. carpio*), some other fish bred there naturally. Those species included Prussian carp (*Carassius gibelio*), tench (*Tinca tinca*), and roach (*Rutilus rutilus*). Each autumn antibiotics (oxytetracycline) were given to the fish, together with feed for the treatment or metahylaxis of bacterial diseases, according to the dosage provided by the manufacturer.

The lake selected for obtaining the wild carp was shallow, (overage depth was 2.5 m) with shallow shores, typical of many lakes of glacial origin located in Lithuania. The predominant fish species within the lake included roach (*Rutilus rutilus*), European perch (*Perca fluviatilis*), common rudd (*Scardinius erythrophthalmus*), and some other fresh water species, mostly from the Cyprinidae family. Although the carp breed naturally, there were records about additional carp stocking of the lake during the last two years (2019–2020).

Two groups of fish, each of 10 carp of similar age (3 years old) and weight (1.5 ± 0.25 kg) were formed, without sex determination, and represented aquacultured (*C. carpio* from fish ponds) and wild fish (*C. carpio* from a lake) populations. The captured fish were brought to the laboratory in plastic bags within 2 h and kept alive in fiberglass tanks. Total length and weight were measured for each fish, whereas the age was determined by a professional ichthyologist. After opening abdominal cavity the intestines were removed. Intestinal content from the distal part of the intestines (0–20 cm from anus) was taken for further molecular and bacteriological testing. Skin samples were taken using sterile cotton swabs from the head area and from the scales of the flanks.

### 2.2. Metagenomic Analysis of Fish Microbiomes

Laboratory testing was performed at the Microbiology and Virology Institute, Lithuanian University of Health Sciences. Cloacal and intestinal contents were collected using 2.0 mL DNAase-free Eppendorf tubes. DNA was extracted using a Quick-DNA Fecal/Soil Microbe Kit (Zymo Research, Irvine, CA, USA) and thereafter concentrated using a Clean and Concentrator-25 Kit (Zymo Research, Irvine, CA, USA), according to manufacturer instructions. Initial quantity and quality of the DNA was controlled using a Nano Drop 2000 (Thermo Fisher, Waltham, MA, USA) spectrophotometer. The samples then were pooled into two separate samples representing the total DNA from the aquacultured and wild carp. Next, 16S metagenomic libraries were prepared, sequenced, quality controlled, and assembled in an independent service laboratory (Baseclear, Leiden, The Netherlands). Short paired sequence reads were generated using an Illumina MiSeq system (Illumina, San Diego, CA, USA) and converted into FASTQ files using the BCL2FASTQ pipeline software, version 1.8.3 (Illumina, San Diego, CA, USA). Subsequently, reads containing a PhiX control signal were removed using an in-house filtering protocol. In addition, reads containing (partial) adapters were clipped up to a minimum read length of 50 bp. The second quality assessment was based on the remaining reads using the FASTQC quality control tool, version 0.11.5. Subsequently, the Illumina paired reads were merged into single reads (so-called “pseudoreads”) through sequence overlap. Chimeric pseudoreads were removed, and the remaining reads were aligned to a combination of the GreenGenes and RDP 16S gene databases. Based on the alignment scores of the pseudoreads, taxonomic classes were assigned by associating each pseudoread to the best matching operational taxonomic unit (OTU).

### 2.3. Bacteriological Testing and Detection of Antimicrobial Resistance

Intestinal and skin sampling from each fish was performed using cotton swabs (Transwab gel Amies gel medium, MWE, Wales, UK) for the isolation of Enterobacteriaceae, *Aeromonas* and *Acinetobacter,* as bacterial indicators for the testing of antimicrobial susceptibility. For isolation of Enterobacteriaceae, MacConkey Agar (Liofilchem, Roseto degli Abruzzi, Italy) was used. Colorex Acinetobacter chromogenic medium (PP3032, EO Labs, Scotland, UK) was used for isolation of *Acinetobacter* spp. For the isolation of *Aeromonas* spp., chromogenic Aeromonas Agar (PP2151, EO Labs, Scotland, UK) was used. The media were incubated at 24 °C for 48 h. One colony of presumptive genus was taken from each sample and from each media. If different colonies were found to be growing, then more than one colony was taken, with the ultimate aim of obtaining more isolates on the target. In order to confirm family and genera, cytochrome oxidase production, as well as other classical biochemical tests were performed, including production of catalase, decomposition of carbohydrates, OF tests, and motility test. The isolates of Enterobacteriaceae were further identified using a Microbact 24E (Thermo Scientific, Loughborough, UK) biochemical identification kit. Antimicrobial susceptibility testing was carried out by the disk diffusion method, according to the EUCAST guidelines and breakpoints. The antimicrobials for testing were selected according to their importance for human and animal medicine with the aim of assessing the potential risk of possible resistance transfer from fish microbiota to the environment. Discs with concentrations recommended by the EUCAST representing different antimicrobial classes, including extended spectrum penicillins, cephalosporins, carbapenems, aminoglycosides, tetracyclines, fluoroquinolones, sulphonamides, and trimethoprim, were used.

### 2.4. Statistical Analysis

Differences between the most prevalent bacteria of separate taxons among two groups of carp from two different environments were assessed using the Z-Test Calculator for two population proportions [8]. The total number of bacterial reads, i.e., the amount of bacteria in a fish gut as discrete variables were compared between the groups as well. All bacterial phyla and classes were compared according to the number within the groups, whereas in lower taxonomic ranks comparison of the most prevalent bacteria (in case of the prevalence being ≥1% of the total amount of bacteria) was performed. Results were considered statistically significant at *p* ≤ 0.05.

## 3. Results

### 3.1. Microbial Profiles in Fish

Two groups, each of 10 carp, represented the aquacultured and wild fish populations. Microbial profiles between the groups, based on 16S rRNA sequencing were compared. Although a good quality of DNA was obtained from both samples the number of reads differed significantly: 33,782 and 67,093 reads were obtained from *C. carpio* from fish ponds and from *C. carpio* from a lake, respectively.

**Figure 1:**
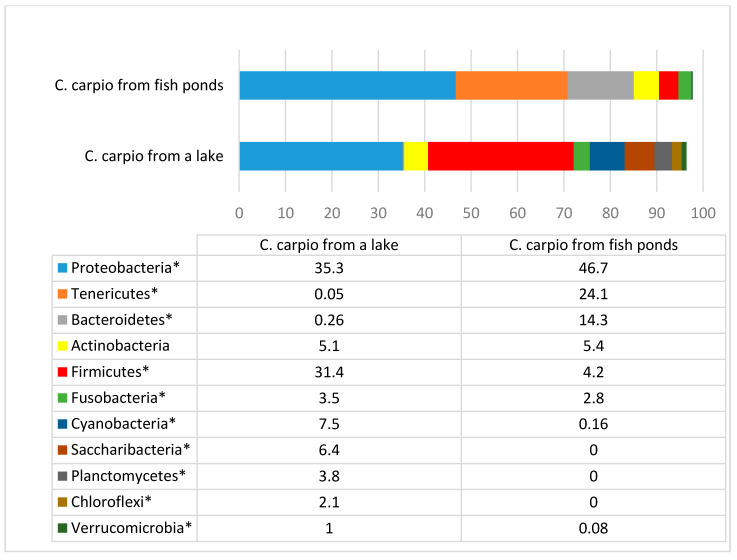
Bacterial phyla prevalence (%) in *Cyprinus carpio* gut. * statistically significant results (*p* ≤ 0.05) between the number of bacteria of separate phyla.

**Figure 2:**
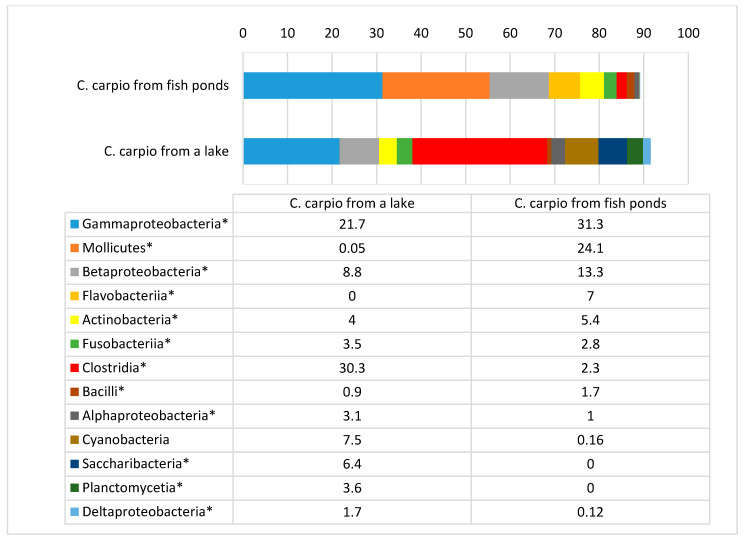
Bacterial class prevalence (%) in *C. carpio* gut. * statistically significant results (*p* ≤ 0.05) between the number of bacteria of separate phyla.

**Figure 3:**
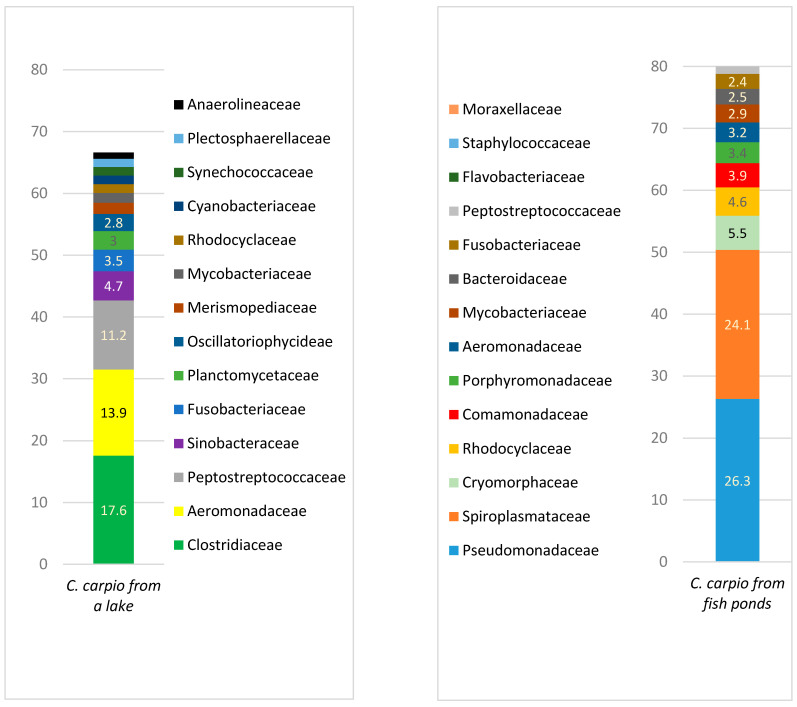
Bacterial family prevalence (%) in *C. carpio* gut (prevalence of at least ≥1%).

**Figure 4:**
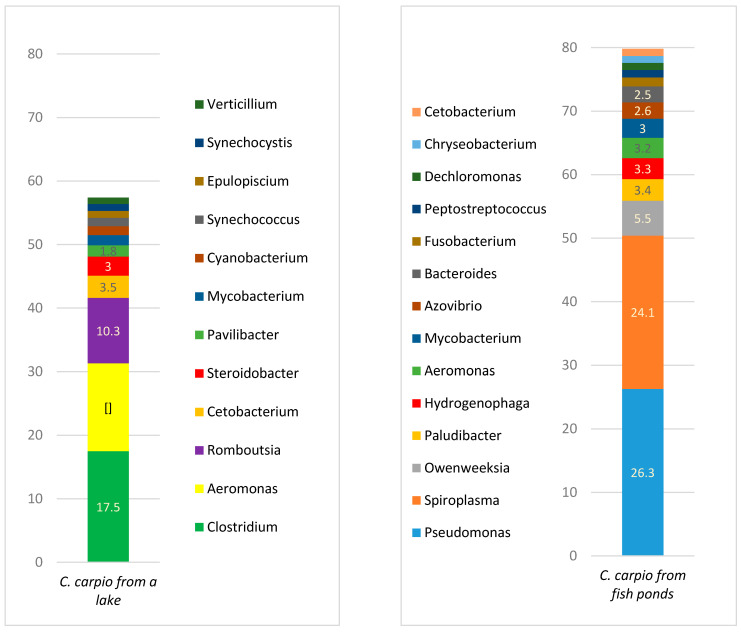
Bacterial genera prevalence (%) in *C. carpio* gut (prevalence of at least ≥1%).

**Figure 5:**
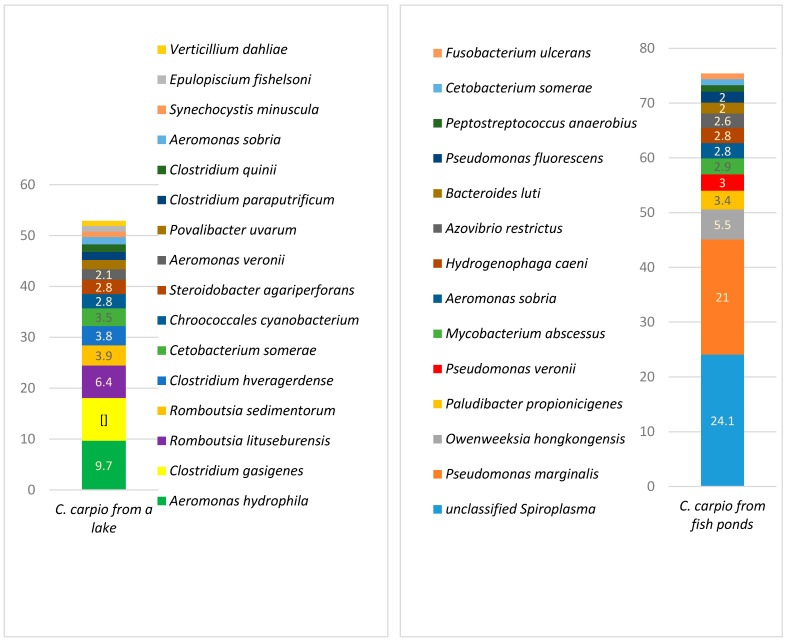
Bacterial species prevalence (%) in *C. carpio* gut (prevalence of at least ≥1%)**.**

A comparison of the microbiota among different taxons in the carp gut is presented in Figure 1, Figure 2, Figure 3, Figure 4 and Figure 5.

As can be seen from Figure 1, obvious differences in the composition of microbiota in both carp groups were observed, even at a phylum level. The differences of bacterial amount in all phyla, except for Actinobacteria, were statistically significant. The main differences were observed among Tenericutes and Bacteroidetes, which were more prevalent in carp from fish ponds, as well as among Firmicutes which were more obvious in fish living in the lake. Some other phyla such as Saccharibacteria, Plamctomycetes, and Chloroflexi were detected only in the gut of the wild carp. The amount of Cyanobacteria and Verrucomicrobia was higher in carps from the lake.

Statistically significant differences were detected among the number of bacteria in all bacterial classes in carp from different environments (Figure 2). The most obvious differences were detected for Mollicutes and Clostridia, which were the most prevalent in fish from ponds and the lake, respectively. The class diversity was higher in the gut of lake carp, as there were none, or only a low number, of the bacteria classes Alphaproteobacteria, Cyanobacteria, Saccharibacteria, Plantomycetia, and Deltaproteobacteria detected in pond fish gut. No Flavobacteria were detected in lake carp, whereas the amount of this class in pond fish was 7% of the total amount of microbiota.

Figure 3 represents microbial profiles from carp gut on a family level. Although the amount of different families with a prevalence above 1% from the total count of bacteria in both groups was similar and contained 14 different families, their taxonomical composition was different. The most prevalent bacterial families in carp from fish ponds were Pseudomonodaceae, Spiroplasmataceae, and Cryomorphaceae, while in the lake carp Clostridiaceae, Aeromonadaceae, and Peptostreptococcaceae predominated. The overall bacterial variety was much higher in lake carp (including those with a prevalence less than 1%) if all the bacterial families detected were counted (Tables S1 and S2). Families which are treated as pathogens or opportunistic pathogens, such as Moraxellaceae, Flavobacteriaceae, and Staphylococcaceae, were detected only in the gut of carp from the fish pond.

Figure 4 represents the bacterial variety in the gut of carp from fish ponds and the lake. The most prevalent genera in carp from fish ponds were *Pseudomonas*, *Spiroplasma*, *Owenweeksia,* and *Paludibacter,* while in the lake carp the predominant genera were *Clostridium*, *Aeromonas*, *Romboutsia,* and *Cetobacterium*. The variety of genera was significantly higher in the gut of lake carp, where 310 genera were identified with a prevalence of ≥0.01%, compared to 118 genera in the pond fish (Tables S3 and S4).

Bacterial variety at a species level is presented in Figure 5. The most prevalent species in the gut of carp from fish ponds were unclassified *Spiroplasma*, *Pseudomonas marginalis*, *Owenweeksia hongkongensis,* and *Paludibacter propionicigenes*; the prevalence of those species was more than 50% of all of the bacterial populations. In the lake carp, the most prevalent species included *Aeromonas hydrophila*, *Clostridium gasigenes,* and two *Romboutsia* species: *R. lituseburensis* and *R. sedimentorum*. In *C. carpio* from the lake all bacteria species with a prevalence of ≥1% had an overall prevalence of <60%, meaning that a high variety of species was present within this group. The total number of species in lake carp was 448, with a prevalence of at least 0.01%, whereas in the pond fish, the number of bacterial species with the same level of prevalence was only 175, i.e., 2.56 times less (Tables S5 and S6).

### 3.2. Antimicrobial Resistance Profile

In total, 68 isolates were obtained from the tested fish (Table 1). Species Enterobacteriaceae are presented in Table 2.

The main genera of the family Enterobacteriaceae included *Klebsiella*, *Citrobacter,* and *Enterobacter*. Zoonotic species were also present, and included *Salmonella enterica*, *Shigella sonnei*, and some other opportunistic species.

**Figure 6:**
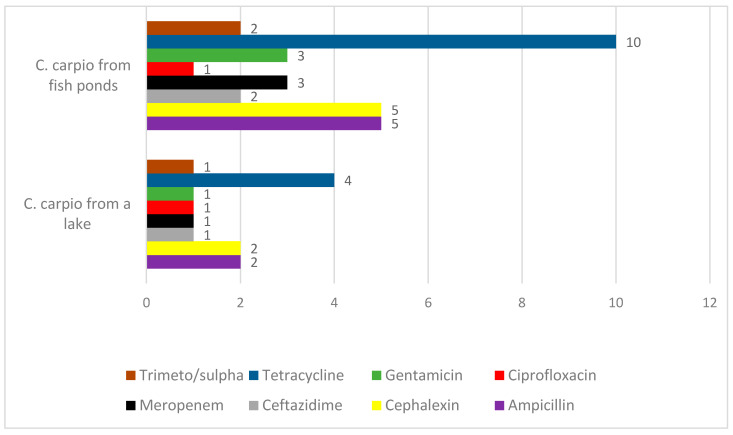
The number of resistant isolates of Enterobacteriaceae isolated from carps.

**Figure 7:**
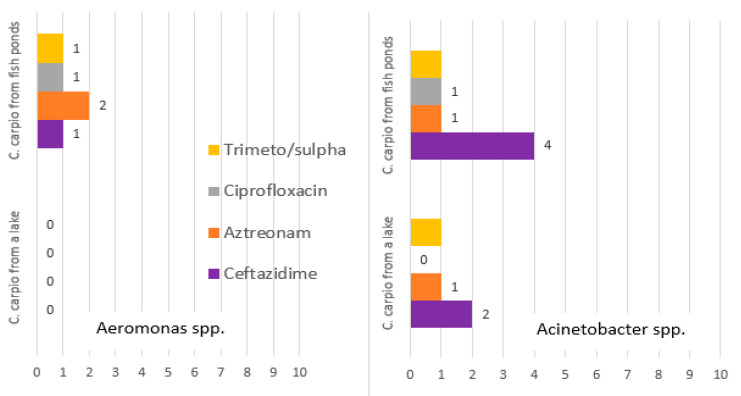
The number of resistant isolates of bacteria genera *Aeromonas* and *Acinetobacter* isolated from carp.

The antimicrobial resistance of the isolates of Enterobacteriaceae, *Acinetobacter* and *Aeromonas,* is presented in Figure 6 and Figure 7.

Bacteria of the family Enterobacteriaceae showed a wide spectrum of resistance to different antimicrobials, including critically important antimicrobials for humans and animals (Figure 6). One isolate resistant to all antimicrobials tested was identified in a wild fish. It was identified as *Klebsiella pneumoniae*. The rest of the isolates demonstrated resistance only to single antibiotics: one isolate was resistant to cephalexin and ampicillin, while three isolates were resistant to tetracycline (25.0%). Isolates obtained from the fish ponds were much more frequently resistant to all classes of antibiotics, except for ciprofloxacin, where only a single isolate was resistant. The most frequent resistances were detected towards tetracycline (83.3%), ampicillin, and cephalexin (41.7%), whereas three isolates were resistant to meropenem and three to gentamicin (25.0%).

Isolates of *Aeromonas* genus from wild carp were susceptible to all antimicrobials tested (Figure 7). The isolates of pond fish in most cases were susceptible, except for two isolates, of which one was resistant to ceftazidime and aztreonam, while the other one, to aztreonam, ciprofloxacin, and sulphamethoxazole/trimethoprim. Antimicrobial resistance profiles of *Acinetobacter* spp. demonstrated more frequent resistance towards meropenem in bacteria isolated from *C. carpio* from fish ponds (40% vs. 20%). Single isolates were resistant to sulphamethoxazole/trimethoprim and ciprofloxacin in both groups. One isolate from pond carp was resistant to gentamicin.

## 4. Discussion

The investigations showed large differences in the composition, and in particular in the variety, of microbiota in the gut of fish from different environments. Different phyla predominated in carp from fish ponds and wild carp. In carp from fish ponds, a high number of Tenericutes and Bacteroidetes were present, whereas in the gut of wild fish Firmicutes were one of the most abundant phyla. The Firmicutes are composed of more than 200 different genera, including *Lactobacillus*, *Bacillus*, *Clostridium*, *Enterococcus,* and *Ruminococcus*; most of which are treated as beneficial bacteria. It is known that *Clostridium* represent 95% of the Firmicute phyla in vertebrates [9] and can be considered part of the normal microbiota in animals. In this case, in lake carps the most prevalent class among Firmicutes was Clostridia. There is a lack of information about the exact role of clostridia in the fish gut, but it is known that bacteria of this class participate as crucial factors in modulating physiologic, metabolic, and immune processes in vertebrates by interacting with the other microbial populations, and also by providing specific and essential functions during digestion processes. They also help prevent dysbiosis [10]. In herbivorous fish, *Clostridium* species have been associated with potentially beneficial roles in vitamin and fatty acid synthesis [11] and the production of metabolic enzymes for catabolism [12]. We detected different species of *Clostridium* in the lake carp, with *C. gasigenes*, *C. hveragerdense*, *C. paraputrificum,* and *C. quinii* among the most prevalent. There is still a lack of information regarding the role of these species in fish.

The phylum Tenericutes in aquacultured fish was associated with high number of bacteria genus *Spiroplasma* from the class Mollicutes. Although the importance of *Spiroplasma* spp. for pond fish remains unclear, it is known that *Spiroplasma* shares a simple metabolism, parasitic lifestyle, fried-egg colony morphology, and small genome, but has a distinctive helical morphology, unlike *Mycoplasma*. It has a spiral shape and moves in a corkscrew motion [13]. Many *Spiroplasma* are found either in the gut or haemolymph of insects, where they can act to manipulate host reproduction, or defend the host as endosymbionts [14]. *Spiroplasma* are also disease-causing agents in the phloem of plants [15], insects [16], and rarely in man [17,18]. It is unclear if bacteria of this genus should be treated as a natural inhabitant of the fish gut or as an incidental finding more associated with the animal’s plant and insect-based diet. Aquacultured fish were fed grain and less frequently with commercial pelleted feed, therefore a high amount (24.1% of a total gut microbiota) of *Spiroplasma* could be associated with fish as a host.

Some studies investigated the possible role of *Spiroplasma* in the pathogenesis of vertebrate-associated neurodegenerative diseases, although no clear evidence that this bacterium is involved in this process has been shown [19]. No data regarding *Spiroplasma* pathogenicity in fish exist; however, recently one species of *Spiroplasma*, *S. eriocheiris*, was identified as a pathogenic species in freshwater crayfish [20].

The prevalence of the bacterial phylum Bacteroidetes was high in aquacultured fish but not in wild fish gut. The main genera from this phylum include *Owenweeksia* from the order Flavobacteriales, in addition to *Paludibacter* and *Bacteroides* from the order Bacteroidales. Both *Paludibacter* and *Bacteroides* are anaerobic bacteria with a fermentative metabolism and have been associated with possibly degrading oligofructose [21]. Some species of Flavobacteriales are potential pathogens and increase the chance of infection in aquaculture animals [22], although currently no information regarding the role of *Owenweeksia* in fish has been reported. The sole species, *O. hongkongensis*, within this genus is known as an “orange-pigmented sea bacterium”, which was first described by Lau et al. in 2005 in marine water [23]. This study shows that *O. hongkongensis* was also presents in the gut of fresh water aquacultured fish.

The variety of bacteria on a genus and species level in aquacultured and wild carp differs significantly. More than 300 different genera were found resident in the gastrointestinal tract in wild carp, whereas in aquacultured fish, only 118 genera were detected, in which the prevalence was at least 0.01% out of the total number of bacteria (which can be treated as autochthonous microbiota of fish). A similar situation was observed for species variety, in which 448 and 175 species were identified in wild and aquacultured carp, respectively. The high diversity of microbial species in fish living in the wild when compared with pond fish can be explained by the larger biological variety in natural water bodies. The levels of flora, insect species, and other invertebrates are much lower in fish ponds, which serve specifically for intensive fish production, continuously within the same place for decades.

The most prevalent bacterial species in wild carp was *Aeromonas hydrophila*. Although this species was previously mentioned as a potential fish pathogen causing erythrodermatitis in carp [24], another species, *A. salmonicida*, is now recognized as an obligatory fish pathogen [25]. We did not detect any signs of diseases by either external examination or during the necropsy of wild carp during this study, or in our previous studies, performed in Lithuania. This finding proves that *A. hydrophila* is a part of the normal skin microbiota of *C. carpio*.

Although the number of isolates tested for antimicrobial susceptibility in this study was quite low, differences between the bacteria in wild and aquacultured fish were detected with respect to antimicrobial resistance. The most frequent resistance was found among the bacterial family, Enterobacteriaceae, and to a lesser extent among the other genera tested. We detected only a single multi-resistant isolate of Enterobacteriaceae isolated from wild carp. The antimicrobial resistance profile included resistance to all classes of tested antimicrobials. We presumed that this isolate was allochthonous, indicating its origin was probably associated with contaminated water from sewage or other waste-water sources. It is known that so called super-bugs, such as the carbapenem- and extended spectrum beta-lactamase-resistant *Klebsella pneumoniae*, as isolated in this case, are widespread throughout health care facilities and are bacteria causing hospital infections [26]. Such multi-resistant bacteria, particularly carbapenemase-producing Enterobacteriaceae, are well-known as widespread nosocomial infectious agents in Lithuania [27]. Frequent resistance to carbapenems was also detected in other bacterial isolates from fish in this study. Therefore, it is important to conduct further investigations about the possibility of the spread of carbapenem-resistant bacteria from healthcare facilities to the general surroundings, and vice-versa, when trying to compare isolates prevalent in fish and nosocomial pathogens. Except for a single isolate from wild fish, it was a clear that bacteria isolated from aquacultured fish were more frequently resistant to all antimicrobials. Antimicrobial resistance of fish microbiota was shown to be more frequent with respect to natural or semi-synthetic antibiotics rather than to synthetic ones. For instance, resistance to fluoroquinolones was detected only in a single isolate, while resistance to cephalexin, ampicillin, and tetracycline was much more frequent. Since fish-associated bacteria are naturally widespread in the aquatic environment, they can be naturally resistant to different antibiotics produced by fungi and bacteria that share the same environment, whereas fluoroquinolones are antimicrobials that are not naturally present in their environment. Our data are in agreement with data obtained by other authors who have detected frequent antimicrobial resistance in bacteria from aquacultured carp [28,29].

The prevalence of antimicrobial resistant bacteria could be an indicator of the antimicrobial treatment of fish. This study proves such a statement, as we detected the most resistance to tetracyclines after oxytetracycline was used for the treatment of the fish.

## 5. Conclusions

Microbiota in aquacultured and wild carp differ in composition and variety, in addition to the frequency of antimicrobial resistance. The main bacterial species prevalent in *C. carpio* gut were *A. hydrophila* (9.7%), *Clostridium gasigenes* (8.4%), and *Romboutsia lituseburensis* (6.4%). The most prevalent species in aquacultured carp were unclassified *Spiroplasma* (24.1%) and *Pseudomonas marginalis* (21%). The number of bacterial species in pond carp was only 39% when compared with carp living in wild conditions. One of the reasons for reduced bacterial variety in aquacultured fish is the prevalence of antimicrobial resistant bacteria, which can better survive under antimicrobial pressure, whereas susceptible bacteria are not capable of doing so. The bacterial family, Enterobacteriaceae, demonstrated the most frequently occurring resistance when compared with the other genera. The most frequent bacterial resistance from this family isolated from pond fish were towards tetracyclines and beta-lactams (83.3% and 41.7%, respectively). A relationship between fish treatment with tetracyclines and frequency of resistance to this antibiotic was observed. This finding may have a negative influence on the environment, including the connecting bodies of water. Reduced bacterial variety among aquacultured fish could also be associated with overall reduced biological variety in fish ponds compared with natural water bodies. Further research is needed to investigate the significance of natural microbiota on fish health and the influence of reduced bacterial variety on the welfare of farmed fish.

## Figures and Tables

**Table 1:** The number of bacterial isolates for antimicrobial resistance testing.

Bacterial Family/Genus	*C. carpio* from a Lake	*C. carpio* from Fish Ponds
Enterobacteriaceae	12	12
Aeromonas	12	12
Acinetobacter	10	10

**Table 2:** Species of Enterobacteriaceae identified from the wild and agricultural carp.

Species and Number of the Isolates	
***C. carpio* from a Lake**	***C. carpio* from Fish Ponds**
*Citrobacter freundii*	*Citrobacter freundii (2)*
*Salmonella enterica*	*Citrobacter galenii (2)*
*Escherichia coli*	*Citrobacter braakii*
*Klebsiella pneumoniae (2)*	*Enterobacter asburiae*
*Klebsiella oxytoca (3)*	*Rahnella aquatilis*
*Cronobacter sakazakii*	*Escherichia coli*
*Enterobacter cloaceae*	*Klebsiella oxytoca (2)*
*Rahnella aquatilis*	*Hafnia alvei*
*Kluyvera cruocrescens*	*Shigella sonnei*

## Data Availability

The data presented in this study are available on request from the corresponding author.

## Supplementary Materials

The following are available online at https://www.mdpi.com/2076-2615/11/4/929/s1, Table S1: Families from lake carps; Table S2: Families from aquacultured carps; Table S3: Genera from lake carps; Table S4: Genera from aquacultured carps; Table S5: Species from lake carps; Table S6: Species from aquacultured carps.
